# Inverted U-Shaped Curvilinear Relationship between Challenge and One's Intrinsic Motivation: Evidence from Event-Related Potentials

**DOI:** 10.3389/fnins.2017.00131

**Published:** 2017-03-28

**Authors:** Qingguo Ma, Guanxiong Pei, Liang Meng

**Affiliations:** ^1^Institute of Neural Management Sciences, Zhejiang University of TechnologyHangzhou, China; ^2^School of Management, Zhejiang UniversityHangzhou, China; ^3^School of Business and Management, Shanghai International Studies UniversityShanghai, China; ^4^Laboratory of Applied Brain and Cognitive Sciences, Shanghai International Studies UniversityShanghai, China

**Keywords:** optimal challenge, intrinsic motivation, flow, self-determination theory, event-related potentials, stimulus-preceding negativity

## Abstract

The balance between task demand and one's competence is critical for the maintenance of intrinsic motivation. According to Flow theory and Self-determination theory, optimal challenge gives rise to the maximum intrinsic motivation, and an inverted U-shaped curvilinear relationship between perceived challenge and one's intrinsic motivation is suggested. In order to provide direct experimental evidences for predictions of these theories, in this study, we employed the two-player StopWatch game that we previously designed, which made references to the game format of a badminton tournament. According to our manipulation, a male participant was defeated by the same-sex player paired with him (played by a well-trained confederate of the experimenter) in two matches, one with a wide margin (the complete defeat condition) and another with a narrow one (the near miss condition). Participants performed better and reported to enjoy the near miss match to a greater extent. Besides, an enlarged Stimulus-preceding negativity was elicited when participants were actively anticipating outcomes in the near miss condition, suggesting greater anticipatory attention toward the outcome and an enhanced intrinsic motivation to win. Thus, converging electrophysiological evidences from this study and our former study confirmed the inverted U-shaped curvilinear relationship between perceived challenge and one's intrinsic motivation.

## Introduction

To most human beings, competitive activities or games, sometimes even dangerous ones, have inherent appeal. Researchers from varied backgrounds have long been working on revealing the attractiveness of these activities. Pioneering investigations on the enjoyment of such activities as rock climbing and chess playing suggested intrinsic motivation to be derived from challenges, which are relatively difficult but are still within the participant's capacities and potential (Csikszentmihalyi, 1975). A recent study reported that outcome uncertainty and suspense accompanied by challenges greatly influenced one's intrinsic motivation. Compared with the game in which one outperformed his or her opponent by a wide margin, the close game led to greater enjoyment and enhanced intrinsic motivation (Abuhamdeh et al., 2015). Combining these results, it is suggested that people tend to get most pleasure in optimally challenging activities, which implies an inverted U-shaped curvilinear relationship between challenge and intrinsic motivation. Accordingly, before the apex of the U-shaped curve is reached, the increasing of one's confronted challenge should lead to increase in the intrinsic motivation level. Afterwards, further increasing of one's confronted challenge should lead to decrease in one's intrinsic motivation instead.

The optimal level (i.e., the apex of the curve) is named as flow in Flow theory, which is described as an intrinsically motivating and fully engaging state of consciousness (Csikszentmihalyi, 1990). Flow theory highlights the importance of optimal challenges for sake of enjoyment, which refers to challenges balanced with one's skills (Csikszentmihalyi, 1975). Specifically, flow's dynamic structure of the perceived match between personal skill and challenge has been divided into three different channels: flow (balanced skill and challenge), boredom (high skill vs. low challenge), and anxiety (low skill vs. high challenge; Csikszentmihalyi and Rathunde, 1993). In fact, the postulate of optimal challenge is consistent with the competence need described in the Self-determination theory (SDT; Deci and Ryan, 1980). In terms of SDT, the basic psychological need of competence is defined as feeling skilled, capable or effective in interactions within a given environment, which is suggested to be a key source of one's intrinsic motivation (Deci and Ryan, 1980, 1985). As a consequence, optimal challenge, compared with a lack of challenge and overwhelming challenge, maximizes one's perceived competence and then enhances one's intrinsic motivation to win.

While mainstream motivation theories give consistent predictions regarding the role of optimal challenge in boosting intrinsic motivation (Csikszentmihalyi, 1975; Deci and Ryan, 1980), direct experimental evidences in support of flow's dynamic structure and more specifically, the inverted U-shaped curvilinear relationship between challenge and intrinsic motivation are still relatively rare. In our previous study (Meng et al., 2016), we developed a two-player StopWatch (SW) game adopting the game format of a badminton tournament. We made sure that a male participant defeated the male confederate of the experimenter (hereafter referred to as “confederate”) paired with him in two matches, one with a wide margin (blowout: the lack of challenge condition) and another with a narrow one (narrow win: the optimal challenge condition). Because intrinsic motivation was difficult to be measured in an objective manner, electroencephalograms (EEGs) were recorded throughout the task and event-related potentials (ERPs) were employed to track one's intrinsic motivation. During the outcome anticipation stage, we observed an enlarged Stimulus-preceding negativity (SPN) in the optimal challenge condition, suggesting that participants formed greater anticipation toward the outcome and were more intrinsically motivated to succeed during close games. To the best of our knowledge, our previous study was the first neuroscientific investigation of Flow theory, which validated the important role of optimal challenge in promoting one's intrinsic motivation (Meng et al., 2016). However, it is worth noting that, our previous study only revealed one side of the coin, as participants finally won in both rounds and felt competent in the end (that is, before the apex of the U-shaped curve is reached). Then what will happen if participants failed in the end and felt incompetent instead? Will perceived challenge still play a role in modulating one's intrinsic motivation in this situation (that is, after the apex of the U-shaped curve has been reached)? This open question remained to be settled.

People often have the experience that they are so close to winning a game or obtaining a reward, only to lose it in the end. In this kind of close game, challenges confronted by people are beyond the skills they obtained by a narrow margin. Compared with completely losing control of the game, this situation may induce a stronger feeling of competence, as well as the experience of flow. Given that optimal challenge is significant for the experience of flow even if people do not manage to win in the end, we would like to compare one's intrinsic motivation between the near miss condition and the complete defeat condition in the controlled laboratory setting. As games are goal directed and competitive in nature (Song et al., 2013), they provide an interesting venue to test our hypotheses. In this study, we engaged participants in the same online SW game derived from Meng et al. (2016), in which each participant was paired with a same-sex confederate. Different from our previous study in which participants won both rounds of the game (Meng et al., 2016), the well-trained confederate won both rounds in this study. In order to manipulate the two challenge levels, the confederate won a round of game with a wide margin while won the other round with a narrow one.

As was done in previous studies (Ma et al., 2014; Jin et al., 2015), EEGs were recorded during the experiment in order to clarify the temporal dynamics of motivational processes. The SPN is a sustained, negative shift in potential that mirrors the anticipation of a motivational stimulus during the pre-feedback period (Brunia, 1988; Böcker et al., 1994; Donkers et al., 2005; Masaki et al., 2010). It typically shows right-hemisphere preponderance and usually maximizes over right prefrontal cortex (for a recent review, see Brunia et al., 2012). Previous studies demonstrated that a larger SPN indicated an enhanced expectation toward the outcome and intensified intrinsic motivation toward the task (e.g., Kotani et al., 2015; Meng and Ma, 2015; Meng et al., 2016; Wang et al., 2017). In the current research, the near miss condition of the game provides optimal challenge compared with the complete defeat condition, which bears more motivational relevance to participants and is predicted to be more intrinsically motivating and engaging. Thus, we assume that participants will form greater anticipation toward feedback during the near miss match, resulting in an enlarged SPN.

A potential limitation of our previous study is that we neglected to collect subjective data, which may serve to support our electrophysiological findings (Meng et al., 2016). To address this limitation, after the formal experiment, participants were asked to report their enjoyment level, effort expended as well as the extent they cared about task performance for each round of the game. Ryan et al. (2006) suggested that one's intrinsic motivation was significantly associated with enjoyment. It was also found that optimal challenge can result in more attention devoted, greater persistence and determination (Aubé et al., 2014). Thus, we predict participants to enjoy more, invest greater effort and care more about task performance in the near miss condition. As to the task performance, previous studies consistently argued that intrinsic motivation plays a significant role in producing adaptive outcomes (e.g., Aubé et al., 2014; Cerasoli and Ford, 2014). For instance, by measuring the perception of time in the computer games, it was found that participants had a greater intrinsic motivation and performed better in the adaptive playing mode than in the overload condition (Keller and Bless, 2008). Thus, we predict participants to be more committed to experimental tasks and to perform better in the near miss condition.

## Methods

### Participants

A total of 20 right-handed male graduate and undergraduate students participated in this study, ranging in age from 20 to 25 years (*M* = 21.75, *SD* = 1.37). They were all native Chinese speakers, had normal or corrected-to-normal vision, and did not have any history of neurological disorder or mental diseases. This research was approved by the Neuromanagement Lab Ethics Committee at Zhejiang University, and all participants provided written informed consent before the experiment. A male experimenter acting as the confederate was matched with male participants as pairs to take part in the formal experiment. Data of two participants were discarded because at least one of the experimental conditions were unsuccessfully manipulated. Thus, there were eighteen valid participants for the final analysis.

### Stimuli and procedure

Before the experiment, the participant was briefly introduced to his co-player (the confederate) face-to-face. Then experimental environment and facilities unfamiliar to participants were introduced. We strictly tracked these procedures to make the participants believe in the authenticity of the two-player online game. After that, the two players were led to take seats in separate rooms which are dim, sound-attenuated and electrically shielded. Experimental stimuli were presented on the computer screen at a distance of 100 cm away from subjects, with a visual angle of 7.50 × 5.40°. Stimuli, recording triggers, and response data were presented and recorded by E-Prime 2.0 (Psychology Software Tools, Pittsburgh, PA, USA). Participants were instructed to use the keypad to complete SW tasks all along.

**Figure 1:**
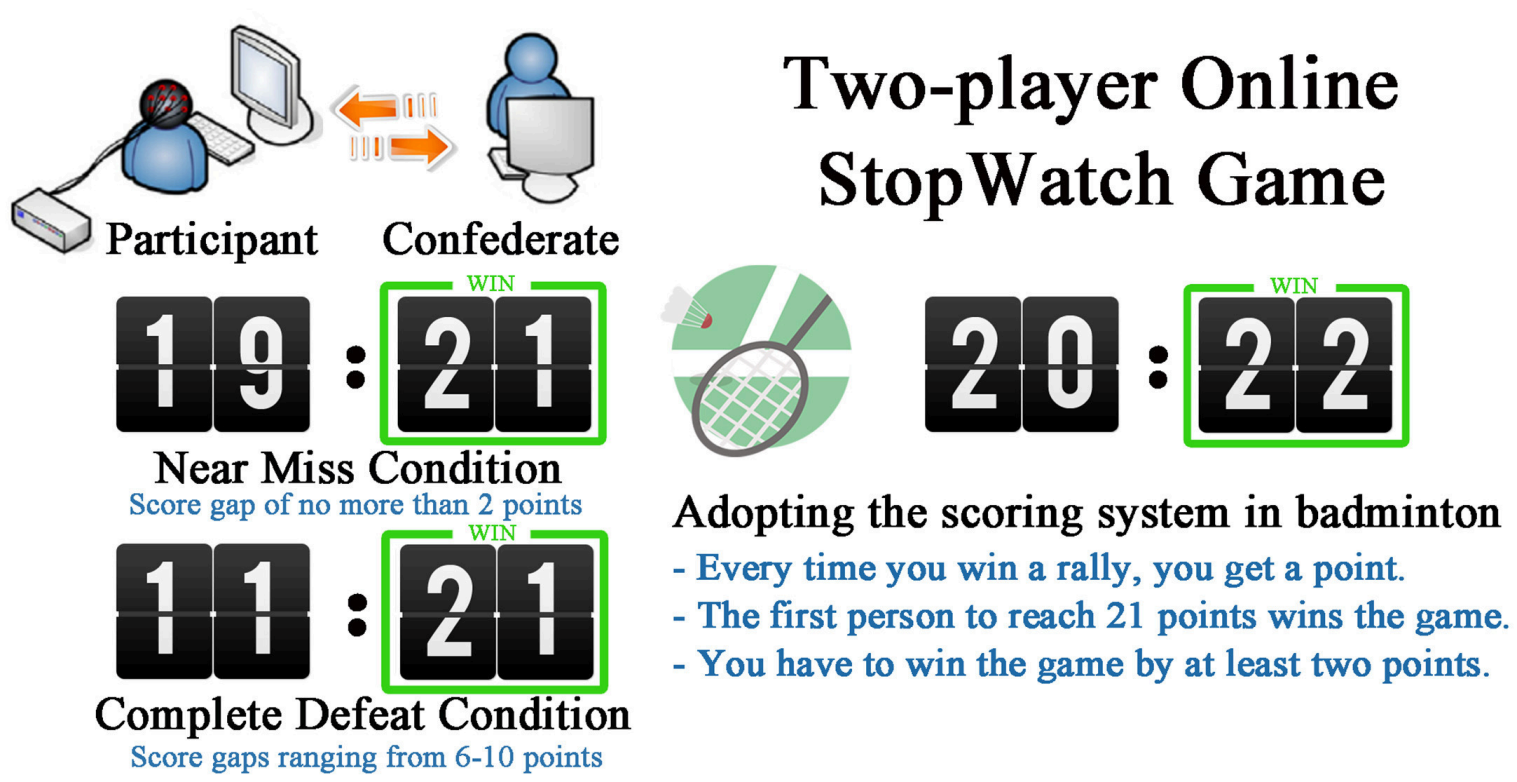
**Game format of the two-player online StopWatch Game**.

SW game was adopted as the experimental task. In the traditional single-player SW game, a stopwatch would automatically start and the player should try his/her best to stop the watch around a specific time point (Murayama et al., 2010; Ma et al., 2014). In one of our previous studies, we designed an interactive online SW game involving two players (Meng et al., 2016). Between the two players, whose response appears closer to 3 s wins the trial and gets one point. Importantly, game format of the badminton tournament was integrated in this game. In other words, the player who accumulates 21 points first and obtains at least 2 more points than his counterpart wins that round of the match. The same rule was adopted in this study (See Figure 1).

**Figure 2:**
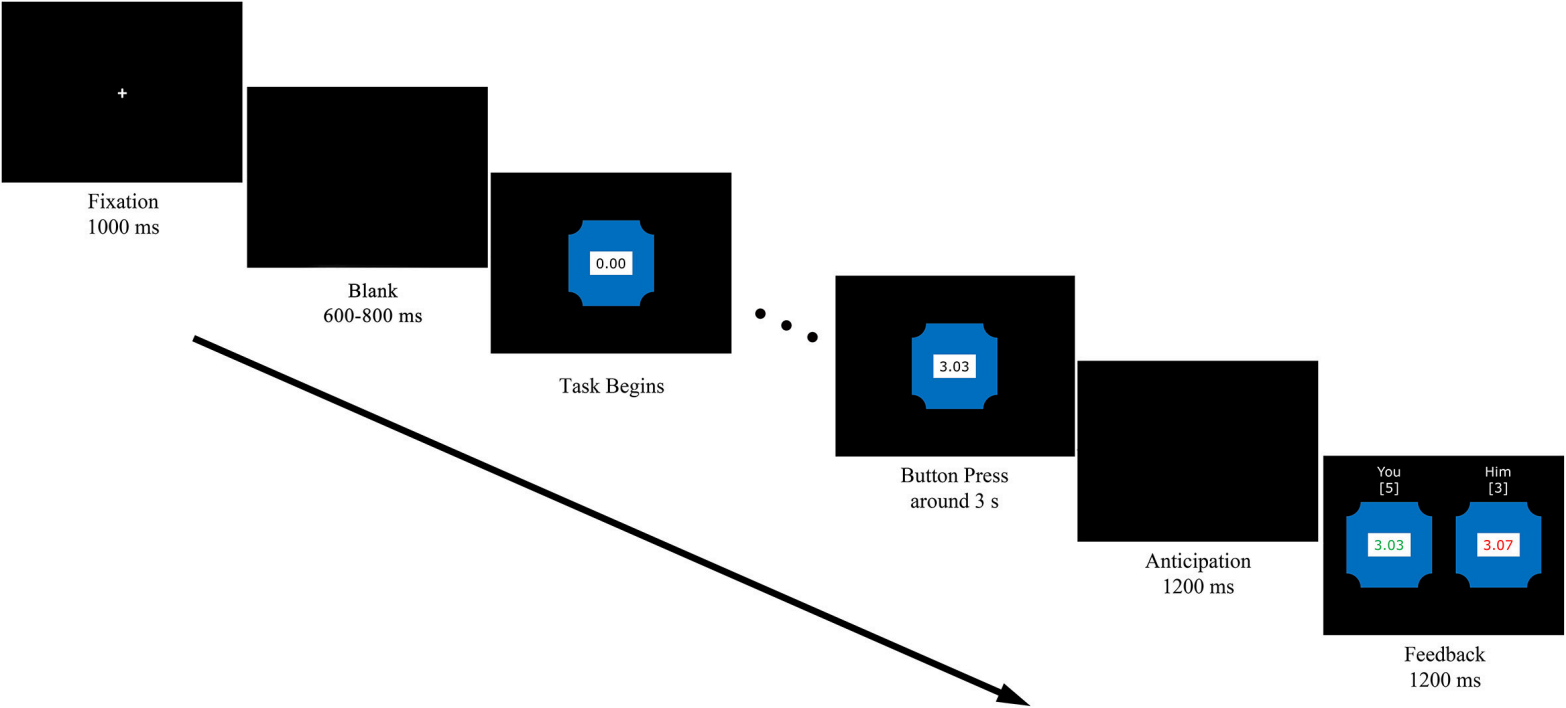
**Illustration of the experimental stimuli applied in a single trial**.

As illustrated in Figure 2, in each trial, a fixation appeared at the very beginning for 1,000 ms. Then, players had to wait for 600–800 ms before the watch automatically started running. After the watch started running, each player could stop it by pressing any of the appointed buttons. If a player pressed the button earlier, he had to wait for the other player to respond. After both players responded, a blank with black background appeared for 1,200 ms. In the end, their performance and the outcome (belongingness of the point) were shown for 1,200 ms. For the player who won, one point would be added, and his performance would be shown in green. The other one would gain no point in this trial and the score would appear in red. If two players responded equally close to 3 s, no one would gain a point and their performance would be shown in black. What's more, the accumulated points would appear on the top of each watch.

For each participant, there were a total of two rounds (blocks) of SW games. During each round, the game would continue until there is a winner. Sequences of experiment conditions (near miss vs. complete defeat) were counterbalanced between participants. For half of them, the first round was set to be a near miss game. Each round of match (a block) contains a minimum of 32 trials (for the complete defeat condition) and a maximum of 50 trials (for the near miss condition). The experimenter who played as the confederate was sufficiently trained and was very good at playing the SW game. In a near miss round, scores of the two players should rise alternatively and the confederate should never accumulate more than 2 points relative to the participant. He should also make sure that the participant lost this round at the last minute. If a round was set to be a complete defeat, the confederate should make sure that he could open the scoring. The score gap gradually widened and the confederate finally won the round with an enormous advantage ranging from 6 to 10 points (as shown in Figure 1). On the extreme case that some of the designs were not successfully manipulated, the data of the involved participant would be discarded. In our study, data of two participants were discarded because of the unsuccessful manipulation.

Prior to the initiation of the formal experiment, each player can practice our single-player version of the SW game for 10 rounds to familiarize themselves with the procedure. They were also informed that they would receive ¥ 35 as compensation for their time and participation, and that their task performance was not related to the final payoff. Thus, participants were intrinsically motivated to win the game rather than to win extra money. After the formal experiment, the participant was asked to report their enjoyment level, effort expended as well as the extent they cared about task performance for each block of the game (from 1 = “the least” to 5 = “the greatest”).

### EEG data acquisition and analysis

EEG was recorded (band-pass 0.05–70 Hz, sampling rate 500 Hz) with Neuroscan Synamp2 Amplifier (Scan 4.3.1, Neurosoft Labs, Inc. Virginia, USA). The elastic electrode cap with 64 Ag/AgCl electrodes was used in accordance with the standard international 10–20 system. A frontal electrode site between FPz and Fz was used as the ground. There were two mastoid electrodes and the left one was used as a reference. Horizontal and vertical electrooculograms (EOG) were monitored with two pairs of electrodes. One pair was located below and above the left eye in parallel with the pupil and the other pair was placed 10 mm from the lateral canthi. The experiment got started only after the electrode impedances were reduced to under 5 kΩ.

In off-line analysis, the EEG data were re-referenced to the algebraically computed average of the left and right mastoids for further analysis. The EOG artifacts with ocular movements were corrected using the method proposed by Miller et al. (1988). The EEGs were digitally low-pass filtered at 30 Hz (24 dB/Octave) and were segmented into epochs from −1,000 ms to feedback onset, with the activity from −1,000 to −800 ms serving as the baseline. Trials that contained amplifier clipping, bursts of electromyography activity, or peak-to-peak deflections that exceeded ±80 μV were excluded from the final averaging procedure. More than 30 sweeps remained for each condition, which are adequate to achieve stable and reliable measurements of typical ERP components (Luck, 2005). The EEG epochs were averaged separately for each condition. Specifically, they were averaged for the complete defeat condition (all trials within the block in which the participant lost with a wide margin) and the near miss condition (all trials within the block in which the participant lost with a narrow margin).

### Statistical analysis

**Figure 3:**
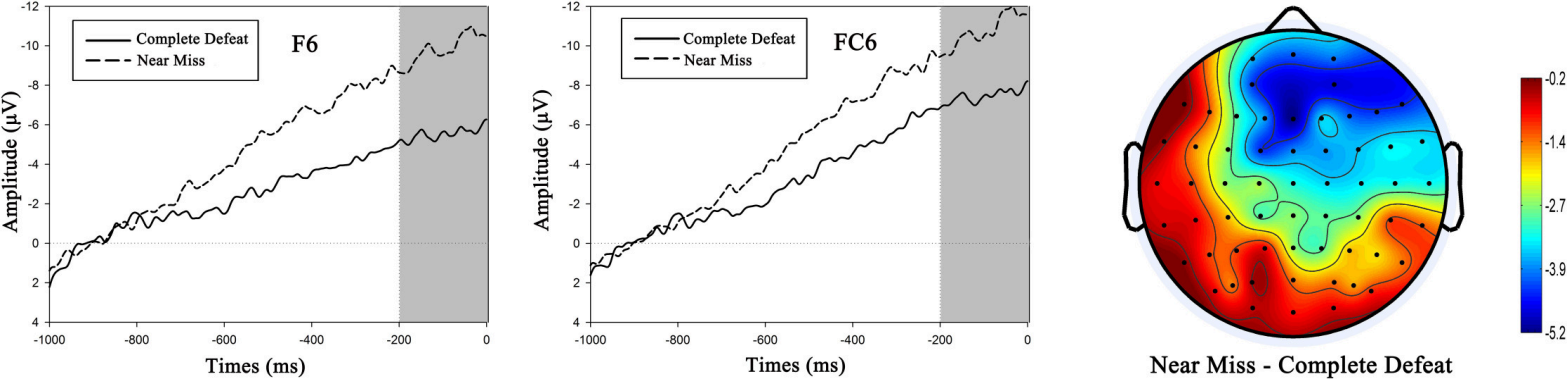
**SPN results during the anticipation stage**. For illustration, grand-average ERP waveforms of SPN from two representative electrodes (F6, FC6) are demonstrated. The scalp topographic distribution of SPN is provided (amplitude in the near miss condition minus that in the complete defeat condition), and the bar ranges from −5.2 to −0.2 μV.

For the behavioral data analysis, since the player whose response was closer to 3 s won the trial, mean absolute deviation around the central point (3 s) was calculated for each condition and a paired *t*-test was used to compare the means. In addition, paired *t*-tests were adopted to compare the means of self-reported enjoyment level, effort expended as well as the extent participants cared about task performance. For the EEG analysis, according to scalp topography of this study (see Figure 3) as well as previous studies (Van Boxtel and Böcker, 2004; Brunia et al., 2011; Meng et al., 2016), SPN has an anterior distribution and typically shows right-hemisphere preponderance. Thus, SPN amplitudes from the electrodes F4, F6, F8, FC4, FC6, and FT8 were analyzed. Mean amplitudes from −200 to 0 ms prior to feedback onset went into a within-subject 2 (challenge level) × 6 (electrode) repeated-measures analysis of variance (ANOVA). The Greenhouse–Geisser correction was applied in all statistical analyses when necessary (Greenhouse and Geisser, 1959).

## Results

### Behavioral results

For the behavioral results, the mean absolute deviation was 0.103 s for the complete defeat condition and was 0.079 s for the near miss condition. The two means were significantly different from each other [*t*_(17)_ = 3.578, *p* = 0.002]. Results of the paired comparisons between subjective data collected from the near miss condition and the complete defeat condition were shown in Table 1.

**Table 1:** **Means of ratings and pair comparisons**.

**Variables**	**Near miss condition**	**Complete defeat condition**	***T*-test results**
Enjoyment	4.056	3.667	*t*_(17)_ = −3.289, *p* = 0.004
Effort	4.667	3.889	*t*_(17)_ = −5.102, *p* < 0.001
Concern about task performance	3.833	3.222	*t*_(17)_ = −4.267, *p* = 0.001

### ERP results

For the 2 (challenge level: near miss vs. complete defeat) × 6 (electrode: F4, F6, F8, FC4, FC6, and FT8) repeated measures ANOVA of SPN, the results showed a significant main effect of challenge level [*F*_(1, 17)_ = 20.315, *p* < 0.001, η^2^ = 0.544]. The near miss condition (*M* ± *SE*, −8.957 ± 0.987 μV) induced a significantly more negative SPN than the complete defeat condition (*M* ± *SE*, −5.488 ± 0.754 μV; as shown in Figure 3). The main effect of electrode was also significant [*F*_(5, 85)_ = 2.601, *p* = 0.031, η^2^ = 0.133]. Nevertheless, the interaction between challenge level and electrode was not significant [*F*_(5, 85)_ = 0.730, *p* = 0.603, η^2^ = 0.041].

## Discussion

Starting from Flow theory and SDT (Csikszentmihalyi, 1975; Deci and Ryan, 1980), this study investigates how the fit between participants' competence and the task demand affects intrinsic motivation. Specifically, intrinsic motivation was examined in the near miss condition and the complete defeat condition of the two-player online SW game. In line with our hypotheses, participants reported to enjoy the near miss round more, and the SPN was more pronounced in the near miss condition.

Pioneering studies demonstrated that SPN reflected the expectancy of a motivational stimulus during the pre-feedback period (Böcker et al., 1994; Donkers et al., 2005; Masaki et al., 2010; Meng and Ma, 2015; Meng et al., 2016; Wang et al., 2017). As the one who has a stronger intrinsic motivation to win would generally care more about the outcome and be more closely anticipating the feedback, and the enhanced subjective expectancy toward feedback would elicit a more prominent SPN, the SPN was suggested as an electrophysiological indicator sensitive to the motivation level (Brunia et al., 2012; Kotani et al., 2015; Meng et al., 2016). In this research, a larger SPN was observed during the feedback anticipation period of the near miss condition. As participants received fixed payoffs irrelevant to task performances, this finding suggested that participants were more intrinsically motivated to win in close games.

According to Flow theory (Csikszentmihalyi, 1975), participants get to experience flow when demands of the task are in balance with their capacities. Too much challenge, which is beyond individuals' capacities, will lead to anxiety and disengagement. In line with Flow theory, in SDT Deci and Ryan (1985) put forward that intrinsically motivated behaviors are partially based in people's needs to feel competent. SDT differentiates the content of outcomes and the processes through which the outcomes are pursued. Although participants lost both rounds of the game, the process in the near miss condition was quite different from that in the complete defeat condition. In the complete defeat condition, the score gap rapidly widened and the participant fell far behind. This dynamic would lead to perceived incompetence, which tends to undermine intrinsic motivation (Deci and Ryan, 2000). It may also lead to weaker self-efficacy and negative mood (Song et al., 2013). As a result, participants were more likely to lose focus and might incline to give up in the end. To the participants, a balanced status was approached between their competence and confronted challenge in the near miss condition relative to the complete defeat condition. Although the participant still fell behind on the whole, they faced a close race with his opponent and their scores rose alternately. Accordingly, they were more likely to be intrinsically motivated and to keep a close eye on their task outcomes throughout the round of the game.

Based on Flow theory and SDT, there may be an inverted U-shaped curvilinear relationship between challenge and intrinsic motivation (Csikszentmihalyi, 1975; Deci and Ryan, 1980). It suggests that increase in challenge should lead to an increase in one's intrinsic motivation up to the apex of the curve, while further increase in challenge would lead to a decrease in one's intrinsic motivation. Meng et al. (2016) suggested the important role of optimal challenge in promoting one's intrinsic motivation to win and provided neural evidence for the validity of the left half of the curve. To be specific, electrophysiological results suggested that participants formed greater anticipatory attention toward feedback and were more intrinsically motivated to win in the narrow win condition compared with during the blowout. In this follow-up study, we gave additional neural evidence for the validity of the curve's right hemisphere (near miss and complete defeat conditions). It is worth noting that dynamics of the narrow win condition in our previous study (Meng et al., 2016) and the near miss condition in this study are highly similar. In both conditions, scores of the participant and the confederate would rise alternatively. The only difference between the two conditions was the belongingness of the winner. Since we tracked the electrophysiological representation of intrinsic motivation during the game (before there is a winner for the round) rather than after the outcome has been determined, the final outcome would not influence one's intrinsic motivation during the game. Thus, both conditions provided optimal challenge to the participants and we deem them to be intrinsically motivated to a similar extent in the two conditions.

**Figure 4:**
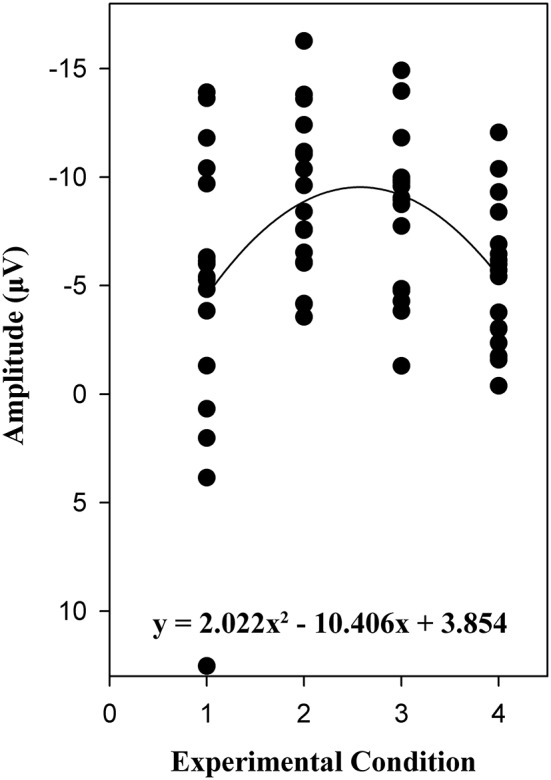
**Verification of the inverted U-shaped model based on electrophysiological results from this study and Meng et al. (2016)**.

Combining electrophysiological results from this study and our previous study (Meng et al., 2016), the inverted U-shaped curvilinear relationship between perceived challenge and one's intrinsic motivation gets validated, and we can conclude that people were indeed most intrinsically motivated in optimally challenging activities. As depicted in Figure 4, challenge is defined as the independent variable, while one's intrinsic motivation is defined as the dependent variable. To be specific, challenge is a nominal variable (1 = blowout, 2 = narrow win, 3 = near miss, and 4 = complete defeat), and we resorted to the mean amplitude of SPN from the six selected electrodes (F4, F6, F8, FC4, FC6, and FT8) to describe one's intrinsic motivation. Through curve fitting, we got an inverted U-shaped fitting curve and a polynomial function (y = 2.022x^2^ − 10.406x + 3.854).

From Figure 4, we can see that only when participants perceived a good match between task skill and confronted challenge (narrow win and near miss conditions) would they experience flow (Csikszentmihalyi and Rathunde, 1993). In fact, similar phenomena can be observed in varied domains. For instance, a cellular level neurophysiological study suggested an inverted-U effect to dopamine D1 receptor (D1R) on prefrontal cortex (PFC) neuronal firing (Vijayraghavan et al., 2007). As a consequence, while spatial working memory gets enhanced by moderate D1R stimulation, it gets impaired by either too little or too much of D1R stimulation.

Across different domains, previous studies have consistently argued that there is a significant role for intrinsic motivation in producing adaptive outcomes (e.g., Keller and Bless, 2008; Aubé et al., 2014; Cerasoli and Ford, 2014). To be specific, it was found that optimal challenge can lead to more attention devoted, greater persistence and stronger determination, all of which are beneficial to the improvement of task performance (Aubé et al., 2014). For instance, Keller and Bless (2008) found that participants in the adaptive playing mode condition reached higher scores on the task than in the overload condition. As a complement to our electrophysiological finding, participants performed better in the near miss condition, and the mean absolute deviation from 3 s in the near miss condition was significantly smaller than that in the complete defeat condition. Besides, participants reported to enjoy the game to a greater extent, to throw more effort into the game and to care more about their task performance in the near miss condition. Taken together, these results suggest that participants feel optimally challenged and more confident in the near miss condition, which make them more committed to experiment tasks even without foreseeable positive results and thus achieve better task performances (Shernoff et al., 2003).

Lab experiments have rigorous restrictions on the experimental design, and it is quite difficult to simulate real-life activities (Meng et al., 2016). However, the design of a two-player online SW task, with a game format similar to the badminton tournament, can not only easily manipulate the challenge level but also arouse participants' inner or “real” feelings in controlled laboratory settings, which is a major contribution of our studies. Besides, this study also has empirical implications. “Gamification”, which applies game elements and design techniques to training and learning programs, has been commonly suggested to effectively facilitate one's enjoyness and flow experience during the target activity (Studer and Knecht, 2016). Adopting this approach, motivation-enhancing elements of the two-player online SW game (e.g., moderate competition) can be elaborately integrated into daily activities. Intrinsic motivation in doing these activities would get strengthened, while original formats of them may keep intact. Another point worth mentioning is that we resorted to ERPs to represent one's intrinsic motivation level. Existing studies on intrinsic motivation mainly relied on established scales. However, self-report itself is highly subjective in nature and may bring observable biases and large variation (Camerer, 2010; Meng et al., 2016). As SPN has been recommended as a neural indicator of one's motivation level (Kotani et al., 2015; Meng and Ma, 2015), we integrated physiological and subjective evidences to achieve a more objective measurement of intrinsic motivation.

## Conclusion

To sum up, in this research we employed the two-player SW game that we previously designed, the game format of which is similar to that of a badminton tournament. Two experimental conditions were cautiously manipulated so that real participants were defeated by a same-sex experimenter with a wide margin in one match (complete defeat) and with a narrow margin in another one (near miss). Converging behavioral and electrophysiological evidences suggested that participants fostered a greater intrinsic motivation to win in the near miss condition. Combined with findings of our previous study (Meng et al., 2016), the inverted U-shaped curvilinear relationship between confronted challenge and one's intrinsic motivation was validated.

## Ethics statement

This study was carried out in accordance with the requirements of the Neuromanagement Lab Ethics Committee at Zhejiang University. All subjects gave a written informed consent according to the Declaration of Helsinki. All participants had normal or corrected-to-normal vision. None of them reported any history of psychiatric or neurological disorders.

## Author contributions

LM and GP conceived and designed the experiment. GP and LM performed the experiment. GP and LM analyzed and interpreted the data. GP, LM and QM wrote and refined the article.

## Funding

QM was supported by grant No. 71371167 from the National Natural Science Foundation of China, grant No. 11ZD028 from Chinese Association of Higher Education, and grant No. AWS12J003 from a national project. LM was funded by “Chen Guang” project supported by Shanghai Municipal Education Commission and Shanghai Education Development Foundation [Grant Number: 16CG36], and a project from the Planning Fund of Shanghai International Studies University [Grant Number: 20161140012].

### Conflict of interest statement

The authors declare that the research was conducted in the absence of any commercial or financial relationships that could be construed as a potential conflict of interest.
